# Nb_2_O_5_ and Ti-Doped Nb_2_O_5_ Charge Trapping Nano-Layers Applied in Flash Memory

**DOI:** 10.3390/nano8100799

**Published:** 2018-10-08

**Authors:** Jer Chyi Wang, Chyuan Haur Kao, Chien Hung Wu, Chun Fu Lin, Chih Ju Lin

**Affiliations:** 1 Department of Electronic Engineering, Chang Gung University, Guishan Dist., Taoyuan 33302, Taiwan; jcwang@mail.cgu.edu.tw (J.C.W.); chiunfu0513@hotmail.com (C.F.L.); yankees0807@hotmail.com (C.J.L.); 2 Department of Neurosurgery, Chang Gung Memorial Hospital, Linkou, Guishan Dist., Taoyuan 33305, Taiwan; 3 Department of Electronic Engineering, Ming Chi University of Technology, Taishan Dist., New Taipei City 24301, Taiwan; 4 Kidney Research Center, Department of Nephrology, Chang Gung Memorial Hospital, Linkou, Guishan Dist., Taoyuan 33305, Taiwan; 5 Department of Electronics Engineering, Chung Hua University, Hsin Chu City 30013, Taiwan; rossiwu@chu.edu.tw

**Keywords:** Ti-doped Nb_2_O_5_, charge trapping nano-layer, MOHOS memory

## Abstract

High-k material charge trapping nano-layers in flash memory applications have faster program/erase speeds and better data retention because of larger conduction band offsets and higher dielectric constants. In addition, Ti-doped high-k materials can improve memory device performance, such as leakage current reduction, k-value enhancement, and breakdown voltage increase. In this study, the structural and electrical properties of different annealing temperatures on the Nb_2_O_5_ and Ti-doped Nb_2_O_5_(TiNb_2_O_7_) materials used as charge-trapping nano-layers in metal-oxide-high k-oxide-semiconductor (MOHOS)-type memory were investigated using X-ray diffraction (XRD) and atomic force microscopy (AFM). Analysis of the C-V hysteresis curve shows that the flat-band shift (∆V_FB_) window of the TiNb_2_O_7_ charge-trapping nano-layer in a memory device can reach as high as 6.06 V. The larger memory window of the TiNb_2_O_7_ nano-layer is because of a better electrical and structural performance, compared to the Nb_2_O_5_ nano-layer.

## 1. Introduction

Conventional polysilicon floating gate issues have been explored in recent years because of problems related to the downscaling of memory devices [1,2,3,4,5]. For this study, high-k materials were chosen as replacements for conventional polysilicon floating gate memory to form metal-oxide-high k-oxide-semiconductor (MOHOS)-type memories [6,7]. Compared with the traditional SONOS-type memory, MOHOS-type has larger memory windows [8,9,10,11]. Otherwise, charge-trapping layers using high-k materials have faster program/erase speeds and better data retention because of larger conduction band offsets and higher dielectric constants [12,13,14,15,16]. Among various rare earth oxides, High-k Nb_2_O_5_ shows a high dielectric constant (k = 40), wide band gap (*E*_G_ = 3.2 eV), and a large conduction band offset [17]. To further enhance the memory performance of the device, addition of Ti atoms into trapping layer has been conducted, and some studies [18,19,20,21,22,23] reported that Ti-doped high-k materials as charge-trapping layers can improve memory device performance such as leakage current reduction, k-value enhancement, program/erase speeds improvement, and charge loss reduction. On the other hand, the performances of other different types of memory structures were presented by previous studies; Ostraat et al. reported that aerosol nanocrystal devices with 0.2 mm channel lengths exhibit large threshold voltage shifts (>3 V), excellent endurance (>10^5^ program/erase cycles), and long-term non-volatility (>10^6^ s) for low-cost non-volatile memory applications [24,25]. Ruffino et al. studied the electrical conduction of Au nanoclusters embedded in SiO_2_ films. The local I-V_tip_ characteristics exhibited an asymmetric behavior with a clear threshold voltage Vth for the electrical conduction, decreasing with the average Au grain size [26].

In our study, this work investigated the differences between metal-oxide-high k-oxide- semiconductor (MOHOS)-type flash memory devices using Nb_2_O_5_ or TiNb_2_O_7_ as charge-trapping nano-layers. Our research indicates that the Ti-doped Nb_2_O_5_ (TiNb_2_O_7_) charge trapping nano-layer has a larger memory window because of the better electrical and structural properties. Therefore, the Ti-doped Nb_2_O_5_-based flash memory might be promising for future industrial memory applications. Structural analyses included X-ray diffraction (XRD) and atomic force microscopy (AFM), while electrical analyses included Capacitance-Voltage (C-V) hysteresis, program/erase speed, and data retention measurements.

## 2. Materials and Methods

**Figure 1:**
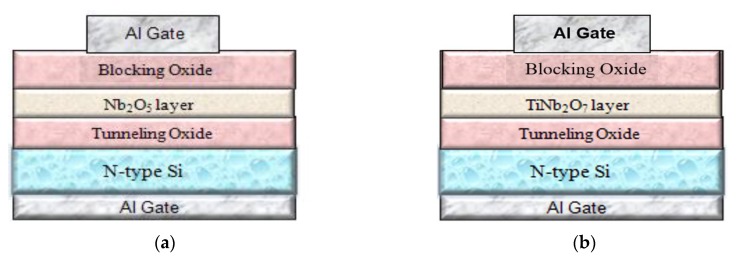
The schematic diagram of (**a**) Al/SiO_2_/Nb_2_O_5_/SiO_2_/Si MOHOS (**b**) Al/SiO_2_/TiNb_2_O_7_/SiO_2_/Si (MOHOS)-type flash memory devices.

The schematic diagrams of Al/SiO_2_/Nb_2_O_5_/SiO_2_/Si and Al/SiO_2_/TiNb_2_O_7_/SiO_2_/Si (MOHOS)-type flash memory devices are shown in Figure 1a,b, respectively. First, single-crystal 4-inch n-type silicon (100) wafers were cleaned using a standard RCA process. Then, a 3-nm SiO_2_ film was thermally grown as a tunneling oxide layer using a dry oxidation furnace system at 850 °C. Then, (a) a 12-nm Nb_2_O_5_ trapping nano-layer was deposited by RF sputtering with a pure niobium target (99.99% pure) in argon (Ar) and oxygen (O_2_) gas ambient, while (b) an approximately 12-nm Ti-doped Nb_2_O_5_ (TiNb_2_O_7_) trapping nano-layer was deposited by RF co-sputtering of a niobium and titanium target as comparison. After that, the above two kinds of samples underwent rapid thermal annealing treatment in O_2_ ambient for 30 second from 700, 800, 900 and 950 °C to form Nb_2_O_5_ and TiNb_2_O_7_ charge trapping nano-layers, respectively. Subsequently, a 20-nm SiO_2_ film was deposited as a blocking oxide layer by plasma-enhanced chemical vapor deposition at a substrate temperature of 300 °C. After deposition of the blocking oxide layer, a 300-nm Al film was deposited by thermal evaporator and a gate pattern was defined through lithography and wet etching. Finally, a 300-nm Al film was deposited on the backside. C-V hysteresis and data retention were measured with an HP-4284 LCR meter, P/E speed was measured by an HP8110 pulse generator, and leakage current was measured using an HP4156C semiconductor parameter analyzer. Structural analyses of the Nb_2_O_5_ and TiNb_2_O_7_ trapping layers were performed by XRD and AFM to examine the connections between electrical characteristics and structural properties. The XRD spectrum was performed by a grazing incidence of CuKa (k = 1.542 A) radiation. The system used a grazing incidence angle (θ = 0.5) in an XRD spectrum in the diffraction angle range (2θ) from 20° to 60°. The surface morphologies of the Nb_2_O_5_ and TiNb_2_O_7_ charge-trapping nano-layers were monitored using atomic force microscopy (AFM) by a Veeco model D5000 operated in tapping mode using an Applied Nano silicon tip with a 50 N/m spring constant. The scan area was 3 um x 3 um with a set engagement ratio of 80%.

## 3. Results and Discussion

**Figure 2:**
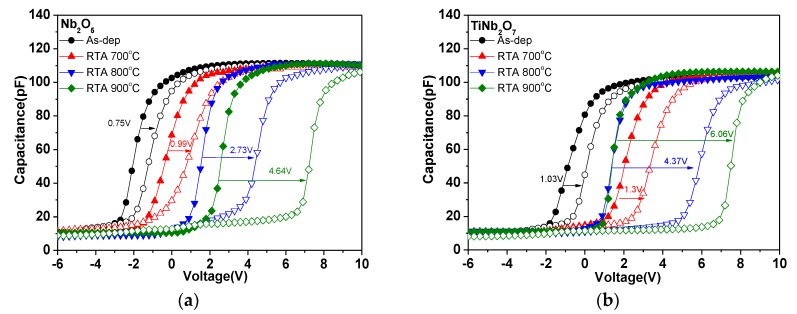
(**a**) High frequency C-V curves of Al/SiO_2_/Nb_2_O_5_/SiO_2_/Si structure after annealing in different temperatures; (**b**) high frequency C-V curves of Al/SiO_2_/TiNb_2_O_7_/SiO_2_/Si structure after annealing in different temperatures.

To examine device performance under different annealing conditions, we compared different C-V hysteresis curves under sweeping voltages, as shown in Figure 2a,b and Figure S1 of the Supplementary Materials. The forward sweep began from inversion region to accumulation and the reverse sweep moved in another direction with the electrons charging into and discharging from the trapping film. The C-V curves indicate that annealing could enlarge the memory window of the device. The Ti-doped Nb_2_O_5_ (TiNb_2_O_7_) sample which underwent RTA treatment at 900 °С had a larger memory window (6.06 V) and better storage capability compared with that of the Nb_2_O_5_ sample annealed at 900 °С (4.64 V). Our research shows that the TiNb_2_O_7_ nano-layer possesses better electrical characteristics than the Nb_2_O_5_ nano-layer, likely because of the enhanced dielectric constants, increased breakdown voltage, and reduced leakage current provided by the addition of Ti. According to the result, it also can be seen that the memory window increased with the annealing temperature from 700 to 900 °С, and the sample annealed at 900 °С had the largest memory window (Nb_2_O_5_: 700 °С/0.99 V, 800 °С/2.73 V, 900 °С/4.64 V; TiNb_2_O_7_: 700 °С/1.3 V, 800 °С/4.37 V, 900 °С/6.06 V). As the material quality of the charge-trapping layer is a decisive factor in trapped electron storage capability, the results indicate that the TiNb_2_O_7_ charge-trapping nano-layer annealing at 900 °С can enhance the formation of a well-crystallized structure.

**Figure 3:**
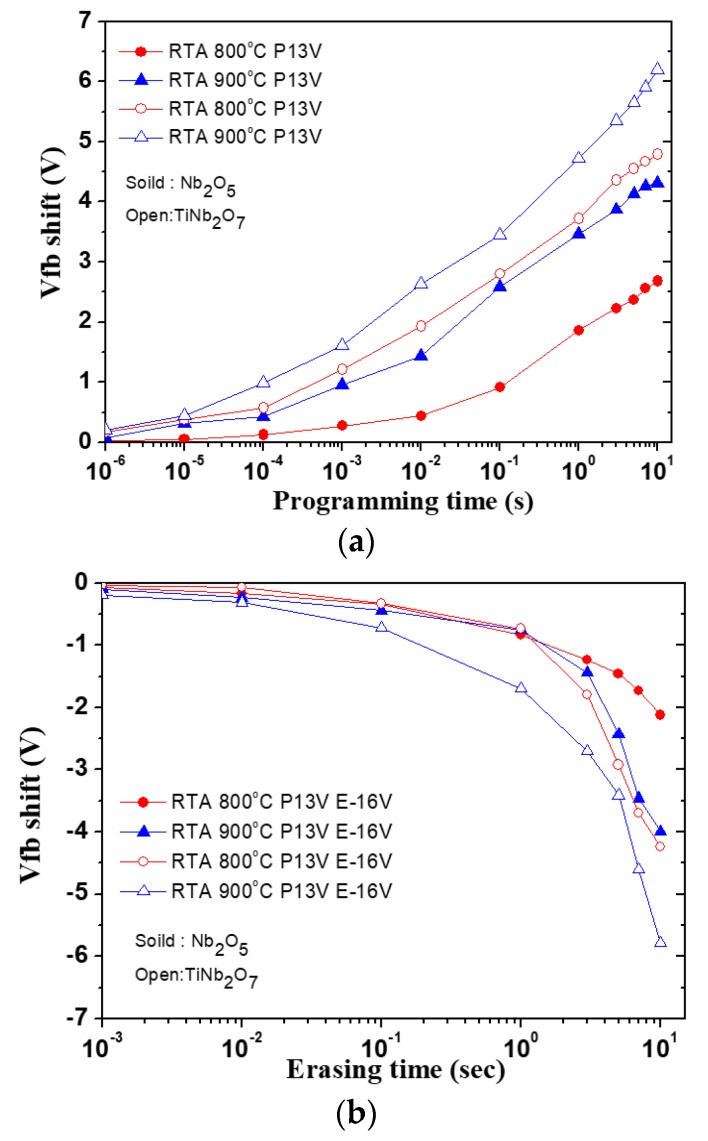
(**a**) Programming and (**b**) erasing speed of the Nb_2_O_5_ and TiNb_2_O_7_ charge trapping nano-layers for 800 and 900 °C annealing under Vg = 13 V programming voltage and −16 V erasing voltage.

Figure 3a,b shows the programming and erasing speed of the Nb_2_O_5_ and TiNb_2_O_7_ charge-trapping nano-layers for 800 and 900 °C annealing under Vg = 13 V programming voltage and −16 V erasing voltage. By the way, the programming and erasing speed of the above samples with applying various bias were also presented in Figures S2 and S3 of the Supplementary Materials. The V_fb_ shift is defined as the change in the flat-band voltage between the virginal and erased states. During supply forward bias, the electron will inject from the Si channel to high-k trapping layers and storage in trapping layers and it will change in fla-band voltage. On the other hand, if we supply reverse bias, the electron will de-trapped from high-k trapping layers to Si channel. In these figures, the programmed state was measured at Vg = 13 V with various time from 1 us to 10 s, and the erase state was measured at Vg = –16 V with various time from 1 ms to 10 s. In our result, it can be found that the TiNb_2_O_7_ charge-trapping nano-layer annealed at 900 °C showed a faster program/erase speed than other samples when operating at same programming/erasing voltage. It can be attributed the TiNb_2_O_7_ charge-trapping nano-layer can enhance higher dielectric constant, which can increase the effective electric field across the tunneling oxide and permit electron easily through FN-tunneling from Si channel to the charge-trapping layers. 

**Figure 4:**
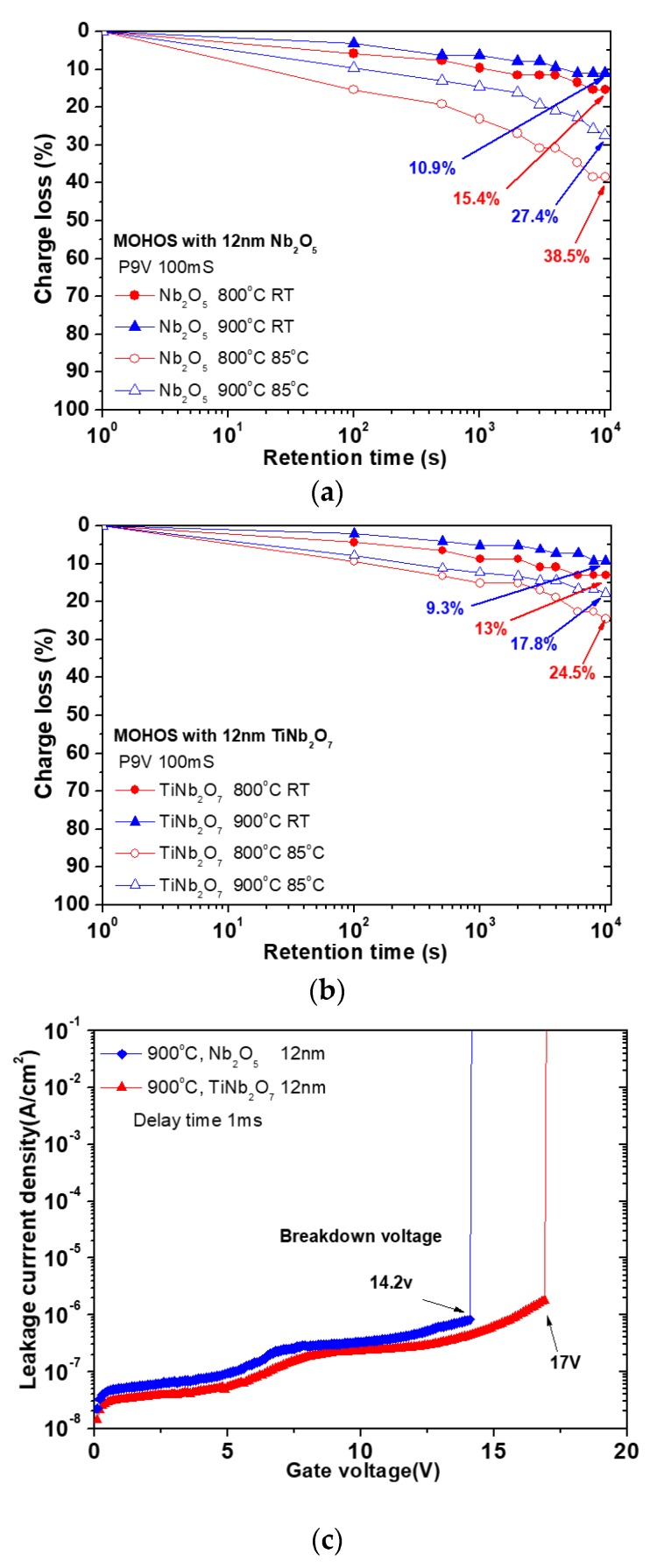
Data retention of the (**a**) Nb_2_O_5_ and (**b**) TiNb_2_O_7_ charge-trapping nano-layers measured at RT and 85 °C; (**c**) the leakage current density versus gate voltage of the Nb_2_O_5_ and TiNb_2_O_7_ trapping nano-layers for the top gate applied a positive bias.

Figure 4a,b shows the retention characteristics of Nb_2_O_5_ and TiNb_2_O_7_ charge-trapping layer after annealing at 800 and 900 °С. The curves were measured at room temperature and 85 °С. The programmed state was the same Vg = 9 V for 100 ms. Charge loss rate was calculated as:

$$Charge\;loss\;rate(\%)=\left[\frac{V_{\left(t\right)}-V_{o}}{V_{1}-V_{o}}\right]\times 100\%$$

where *V*_(*t*)_ is the *V*_fb_ of various time, *V*_1_ is the first *V*_fb_ after being programmed, and *V*_0_ is the fresh one. In our result, the sample annealing at 900 °C has better retention characteristics than that at 800 °C, it’s because of formation structures in high temperature. On the other hand, the MOHOS-type memory with TiNb_2_O_7_ charge-trapping nano-layer after annealed at 900 °C shows a smaller charge loss rate than other samples. The sample annealed at 900 °C shows a smaller charge loss of 9.3% at room temperature (RT) and 17.8% at 85 °C after 1 × 10^4^ s, this is possibly because of the formation of well-crystallized TiNb_2_O_7_ trapping nano-layer. In Figure 4c, shows the leakage current curves of Nb_2_O_5_ and TiNb_2_O_7_ trapping nano-layer, it can be seen that the TiNb_2_O_7_ sample shows a lower leakage current than Nb_2_O_5_ sample, this is because the TiNb_2_O_7_ can reduce the leakage current and enhance the breakdown of the electric field to improve effectively the above charge loss.

**Figure 5:**
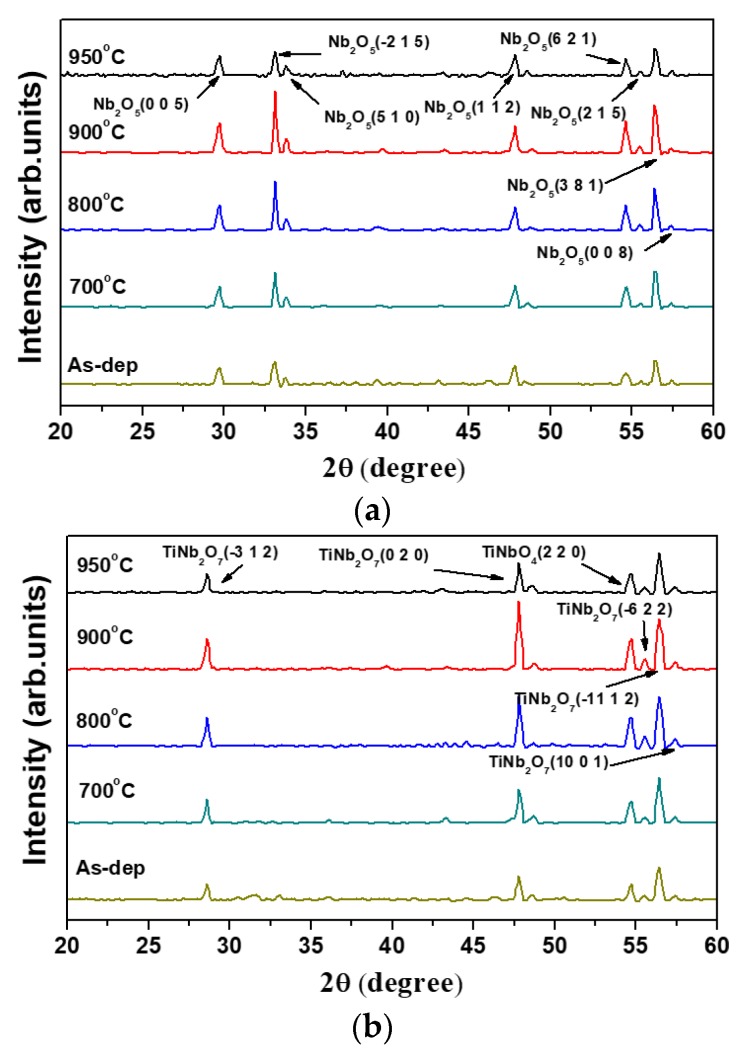
XRD spectra of the (**a**) Nb_2_O_5_ and (**b**) TiNb_2_O_7_ films.

In order to identify the composition of Nb_2_O_5_ and TiNb_2_O_7_ structures at different temperatures, the X-ray diffraction has been used for analysis of Nb_2_O_5_ and TiNb_2_O_7_ structures. The XRD analysis in the range of diffraction angle 2θ from 20° to 60° was obtained by grazing incidence angle (θ = 0.5°) measurements. Figure 5a,b show the XRD analysis of MOHOS-type memory with Nb_2_O_5_ and TiNb_2_O_7_ trapping nano-layer before and after annealing at different temperatures. For the Nb_2_O_5_ nano-layer, it can be seen that the temperature will induce crystallization in high-k trapping layer. According to the XRD analysis, the Nb_2_O_5_ trapping nano-layer has some Nb_2_O_5_ diffraction peaks in (0 0 5), (−2 1 5), (1 1 2), (6 2 1), and (3 8 1) at 29.6°, 33.1°, 47.8°, 54.6°, and 56.4°. The peak intensity became stronger at higher annealing temperatures, and the Nb_2_O_5_ nano-layer annealing at 900 °C exhibited stronger peak intensity in Nb_2_O_5_ (−215), it can be seen that the sample annealing at 900 °C formed a better Nb_2_O_5_ nano-layer structure. For the TiNb_2_O_7_ trapping nano-layer, it has some TiNb_2_O_7_ diffraction peaks in TiNb_2_O_7_ (−3 1 2), TiNb_2_O_7_ (0 2 0), TiNbO_4_ (2 2 0), and TiNb_2_O_7_ (−11 1 2) at 28.6°, 47.8°, 54.7°, and 56.4°. The similar trend that the TiNb_2_O_7_ peak intensity also became stronger at higher annealing temperature and the TiNb_2_O_7_ nano-layer annealing at 900 °C exhibited the strongest peak intensity in TiNb_2_O_7_ (020) than the other temperatures. This means the crystallization enhancement can be found when the sample annealing is at high temperatures. However, when the annealing temperature increased to 950 °C, the TiNb_2_O_7_ peaks declined. This may be because of the formation of a thicker Nb-silicate layer between TiNb_2_O_7_ charge-trapping nano-layer and oxide interface layer to degrade the crystallinity of TiNb_2_O_7_ layer [27]. 

**Figure 6:**
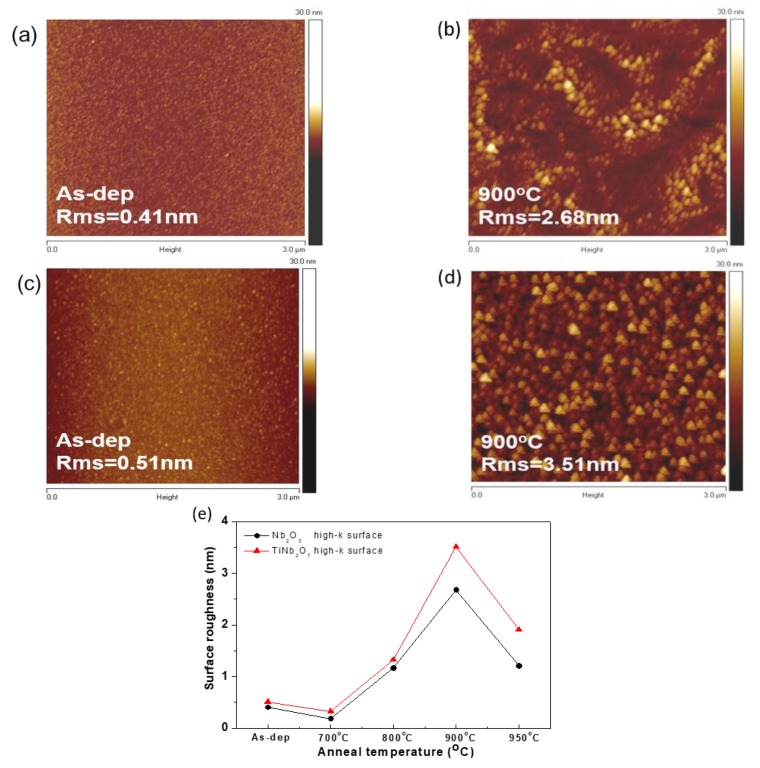
AFM images of the (**a**) as-deposited and (**b**) 900 °C annealed Nb_2_O_5_ films; AFM images of the (**c**) as-deposited and (**d**) 900 °C annealed TiNb_2_O_7_ films; (**e**) surface roughness of Nb_2_O_5_ and TiNb_2_O_7_ films as a function of annealing temperature.

To visualize the film surface texture in a more practical manner, AFM was used to examine surface roughness of the Nb_2_O_5_ and TiNb_2_O_7_ nano-layers for the as-deposited and RTA-annealed samples at 900 °С, as shown in Figure 6a–d. It can be seen that the surface roughness of Nb_2_O_5_ and TiNb_2_O_7_ nano-layers of the as-deposited and 900 °С annealed samples exhibit 0.41 nm, 0.51 nm, 2.68 nm, and 3.51 nm, respectively. Since annealing could effectively increase the surface roughness of the film and reinforce the crystallization of TiNb_2_O_7_ nano-layers, larger roughness values and larger grains could be observed with increasing annealing temperatures. In line with the above results, the roughest surface and largest grains occurred for the TiNb_2_O_7_ nano-layers after RTA treatment at 900 °С. The increase in surface roughness is attributed to the niobium oxide intermixing with the titanium film, resulting in the enhancement of grain growth when annealed at 900 °C. However, the TiNb_2_O_7_ nano-layer annealed at 950 °C exhibited a decrease of roughness as compared to 900 °C, indicating that the oxygen leaving the TiNb_2_O_7_ layer and moving to the TiNb_2_O_7_/oxide interface resulted in a poorly crystallized TiNb_2_O_7_ structure and a low-k interfacial Nb silicate layer at the TiNb_2_O_7_/oxide interface [28]. Figure 6e shows details of the surface roughness of Nb_2_O_5_ and TiNb_2_O_7_ nano-layers, with increasing annealing temperature. Overly, all the samples with Ti-doping show higher surface roughness as compared to Nb_2_O_5_. Larger grains are formed owing to the higher oxygen affinity of Ti increases in the reaction of Nb_2_O_5_ and thus enhances the formation of grain growth to cause rougher surface [29,30]. Moreover, the Ti-doped Nb_2_O_5_ (TiNb_2_O_7_) charge-trapping layer at 900 °C exhibited a larger grain size with more uniform distribution compared with the Nb_2_O_5_ layer at 900 °C. The increase in surface roughness is attributed to the titanium-incorporated niobium oxide film resulting in the enhancement of grain growth to obtain a larger grain size [31,32,33,34]. 

## 4. Conclusions

Our research evaluated Nb_2_O_5_ and Ti-doped Nb_2_O_5_ charge trapping layers for non-volatile memory applications. Compared with the Nb_2_O_5_ trapping layer, the devices with a TiNb_2_O_7_ trapping layer exhibited a larger memory window and faster program/erase speeds. The results indicate that the TiNb_2_O_7_ charge-trapping nano-layer annealing at 900 °С can reinforce the bonding strength to enhance the formation of a well-crystallized structure for performance improvement.

In addition, the TiNb_2_O_7_ trapping layer shows a smaller charge loss of about 9.3% at room temperature and 17.8% at 85 °C after 10^4^ s, because the TiNb_2_O_7_ layer can reduce the leakage current and enhance the breakdown electric field to effectively improve the charge loss. Moreover, structural analyses of the Nb_2_O_5_ and TiNb_2_O_7_ trapping layers were performed by XRD and AFM to investigate and confirm the connections between electrical characteristics and structural properties. Therefore, the MOHOS-type memory device with the Ti-doped Nb_2_O_5_ charge-trapping nano-layer shows itself to be a very promising candidate for future non-volatile flash memory.

## Supplementary Materials

The following are available online at http://www.mdpi.com/2079-4991/8/10/799/s1, Figure S1: (a) Nb_2_O_5_ and (b) TiNb_2_O_7_ charge trapping nano-layers for different temperature after applying various bias; Figure S2: Programming speed of Nb_2_O_5_ trapping layer for (a) 800 °C, and (b) 900 °C after applying various bias, and programming speed of TiNb_2_O_7_ trapping layer for (c) 800 °C, and (d) 900 °C after applying various bias; Figure S3: Erasing speed of Nb_2_O_5_ trapping layer for (a) 800 °C, and (b) 900 °C after applying various bias, and erasing speed of TiNb_2_O_7_ trapping layer for (c) 800 °C, and (d) 900 °C after applying various bias.

## References

[1] Kahng D., Sze S.M. (1967). A floating gate and its application to memory devices. IEEE Trans. Electron. Dev..

[2] White M.H., Adams D.A., Bu J. (2000). On the go with SONOS. IEEE Circuits Devices Mag..

[3] Kim J.H., Choi J.B. (2004). Long-Term Electron Leakage Mechanisms through ONO Interpoly Dielectric in Stacked-Gate EEPROM Cells. IEEE Trans. Electron. Dev..

[4] Bu J., White M.H. (2002). Retention reliability enhanced SONOS NVSM with scaled programming voltage. IEEE Aerosp. Conf..

[5] Swift C.T., Chindalore G.L., Harber K., Harp T.S., Hoefler A., Hong C.M., Ingersoll P.A., Li C.B., Prinz E.J., Yater J.A. An embedded 90 nm SONOS nonvolatile memory utilizing hot electron programming and uniform tunnel erase. Proceedings of the Electron Devices Meeting.

[6] Wang X., Kwong D.-L. (2006). A novel high-κ SONOS memory using TaN/Al_2_O_3_/Ta_2_O_5_/HfO_2_/Si structure for fast speed and long retention operation. IEEE Trans. Electron. Dev..

[7] Wang X., Liu J., Bai W., Kwong D.-L. (2004). A novel MONOS-type nonvolatile memory using high-κ dielectrics for improved data retention and programming speed. IEEE Trans. Electron. Dev..

[8] Hsu T.-H., You H.-C., Ko F.-H., Lei T.-F. (2006). PolySi-SiO_2_-ZrO_2_-SiO_2_-Si flash memory incorporating a sol-gel-derived ZrO_2_ charge trapping layer. J. Electrochem. Soc..

[9] Lee C.-H., Hur S.-H., Shin Y.-C., Choi J.-H., Park D.-G., Kim K. (2005). Charge-trapping device structure of SiO_2_/SiN/high-k dielectric Al_2_O_3_ for high-density flash memory. Appl. Phys. Lett..

[10] Yang S.-M., Chien C.-H., Huang J.-J., Lei T.-F., Tsai M.-J., Lee L.-S. (2007). Cerium oxide nanocrystals for nonvolatile memory applications. Appl. Phys. Lett..

[11] You H.-C., Hsu T.-H., Ko F.-H., Huang J.-W., Yang W.-L., Lei T.-F. (2006). SONOS-type flash memory using an HfO_2_ as a charge trapping layer deposited by the sol-gel spin-coating method. IEEE Electron. Device Lett..

[12] Tan Y.N., Chim W.K., Cho B.J., Choi W.K. (2004). Over-Erase Phenomenon in SONOS-Type Flash Memory and its Minimization Using a Hafnium Oxide Charge Storage Layer. IEEE Trans. Electron. Dev..

[13] Specht M., Reisinger H., Stadele M., Hofmann F., Gschwandtner A., Landgraf E., Luyken R.J., Schulz T., Hartwich J., Dreeskornfeld L. Retention time of novel charge trapping memories using Al_2_O_3_ dielectrics. Proceedings of the 33rd European Solid-State Device Research Conference.

[14] Tan Y.N., Chim W.K., Choi W.K., Joo M.S., Ng T.H., Cho B.J. High-k HfAlO charge trapping layer in SONOS-type nonvolatile memory device for high speed operation. Proceedings of the IEEE International IEDM Technical Digest.

[15] Sugizaki T., Kohayashi M., Ishidao M., Minakata H., Yamaguchi M., Tamura Y., Sugiyama Y., Nakanishi T., Tanaka H. Novel multi-bit SONOS type flash memory using a high-κ charge trapping layer. Proceedings of the 2003 Symposium on VLSI Technology.

[16] Pan T.M., Yeh W.W. (2008). A high-k Y_2_O_3_ charge trapping layer for nonvolatile memory application. Appl. Phys. Lett..

[17] Soares M.R.N., Leite S., Nico C., Peres M., Fernandes A.J.S., Graca M.P.F., Monteiro R., Monteiro T., Costa F.M. (2011). Effect of processing method on physical properties of Nb_2_O_5_. J. Eur. Ceram. Soc..

[18] Van Dover R.B. (1999). Amorphous lanthanide-doped TiOx dielectric films. Appl. Phys. Lett..

[19] Kao C.H., Chen C.C., Huang C.Y., Lin C.J., Ou J.C. (2012). Investigation of Ti-doped Gd_2_O_3_ charge trapping layer with HfO_2_ blocking oxide for memory application. Thin Solid Films.

[20] Chen F.H., Pan T.M., Chiu F.C. (2011). Metal–Oxide–High-k-Oxide–Silicon Memory Device Using a Ti-Doped Dy_2_O_3_ Charge-Trapping Layer and Al_2_O_3_ Blocking Layer. IEEE Trans. Electron. Devices.

[21] Schroeder T., Lupina G., Dabrowski J., Mane A., Wenger C., Lippert G., Müssig H.-J. (2005). Titanium-added praseodymium silicate high-k layers on Si (001). Appl. Phys. Lett..

[22] Kao C.H., Chen H., Chen S.Z., Hung S.-H., Chen C.Y., He Y.-Y., Lin S.-R., Hsieh K.-M., Lin M.-H. (2015). Effects of annealing on CeO_2_-based flash memories. Vacuum.

[23] Kao C.H., Chen H., Chen C.C., Chen C.P., Wang J.J., Chen C.Y., Chen Y.T., Lin J.H., Chu Y.C. (2015). Comparison of electrical and physical characteristics between Gd_2_O_3_ and Ti-doped GdTixOy trapping layers. Microelectron. Eng..

[24] Ostraat M.L., De Blauwe J.W., Green M.L., Bell L.D., Brongersma M.L., Casperson J., Flagan R.C., Atwater H.A. (2001). Synthesis and characterization of aerosol silicon nanocrystal nonvolatile floating-gate memory devices. Appl. Phys. Lett..

[25] De Blauwe J. (2002). Nanocrystal nonvolatile memory devices. IEEE Trans. Nanotechnol..

[26] Ruffinoa F., Grimaldi M.G., Giannazzo F., Roccaforte F., Raineri V. (2006). Nanoscale voltage tunable tunnel rectifier by gold nanostructures embedded in SiO_2_. Appl. Phys. Lett..

[27] Pan T.-M., Chen F.-H., Jung J.-S. (2010). A high-k Tb_2_TiO_5_ nanocrystal memory. Appl. Phys. Lett..

[28] Pan T.-M., Yu T.-Y. (2008). Silicon-oxide-nitride-oxide-silicon-type flash memory with a high-k NdTiO_3_ charge trapping layer. Appl. Phys. Lett..

[29] Van Hal R.E.G., Eijkel J.C.T., Bergveld P. (1996). A general model to describe the electrostatic potential at electrolyte oxide interfaces. Adv. Colloid Interface Sci..

[30] Pan T.-M., Yu T.-Y., Wang C. (2008). High-k Nd_2_O_3_ and NdTiO_3_ Charge Trapping Layers for Nonvolatile Memory Metal-SiO_2_-High-k-SiO_2_-Silicon Devices. J. Electrochem..

[31] Ruffino F., Grimaldi M.G. (2010). Atomic force microscopy study of the growth mechanisms of nanostructured sputtered Au film on Si(111): Evolution with film thickness and annealing time. J. Appl. Phys..

[32] Palasnatzas G., Krim J. (1994). Scanning Tunneling Microscopy Study of the Thick Film Limit of Kinetic Roughening. Phys. Rev. Lett..

[33] Ruffino F., Grimaldi M.G., Giannazzo F., Roccaforte F., Raineri V. (2009). Atomic Force Microscopy Study of the Kinetic Roughening in Nanostructured Gold Films on SiO_2_. Nanoscale Res. Lett..

[34] Chevrier J., Le Thanh V., Buys R., Derrien J. (1991). A RHEED Study of Epitaxial Growth of Iron on a Silicon Surface: Experimental Evidence for Kinetic Roughening. Europhys. Lett..

