# Supplementary material for: Nb2O5 and Ti-Doped Nb2O5 Charge Trapping Nano-Layers Applied in Flash Memory
Source: Nanomaterials (Basel). 2018 Oct 8;8(10):799. doi: 10.3390/nano8100799 (PMC6215173; doi:10.3390/nano8100799)
Supplement: Supplementary file 1 [file nanomaterials-08-00799-s001.pdf]

Supplementary Materials for

Nb_2O_5 and Ti-doped Nb_2O_5 Charge Trapping Nano-Layers Applied in Flash Memory

Jer Chyi Wang[1,3,5], Chyuan Haur Kao[1, 4,5,*], Chien Hung Wu[2], Chun Fu Lin[1], and Chih Ju Lin [1]

[1] Department of Electronic Engineering, Chang Gung University, Guishan Dist., Taoyuan 33302, Taiwan;

[2]Department of Electronics Engineering, Chung Hua University, Hsin Chu City 30013, Taiwan;

[3] Department of Neurosurgery, Chang Gung Memorial Hospital, Linkou, Guishan Dist., Taoyuan 33305, Taiwan

[4] Kidney Research Center, Department of Nephrology, Chang Gung Memorial Hospital, Linkou, Guishan Dist., Taoyuan 33305, Taiwan

[5] Department of Electronic Engineering, Ming Chi University of Technology, Taishan Dist., New Taipei City 24301, Taiwan

*** Correspondence: chkao@mail.cgu.edu.tw; Tel.: +886-3-2118800 (ext. 5783)***

Contents:

Figure S1. (a) Nb_2O_5 charge trapping nano-layers for different temperature after applying various bias.
(b) $TiNb_2O_7$ charge trapping nano-layers for different temperature after applying various bias.

Figure S2 Programming speed of Nb_2O_5 trapping layer for (a) 800°C, and (b) 900°C after applying various bias.
Programming speed of $TiNb_2O_7$ trapping layer for (c) 800°C, and (d) 900°C after applying various bias.

Figure S3 Erasing speed of Nb_2O_5 trapping layer for (a) 800°C, and (b) 900°C after applying various bias.
Erasing speed of $TiNb_2O_7$ trapping layer for (c) 800°C, and (d) 900°C after applying various bias.

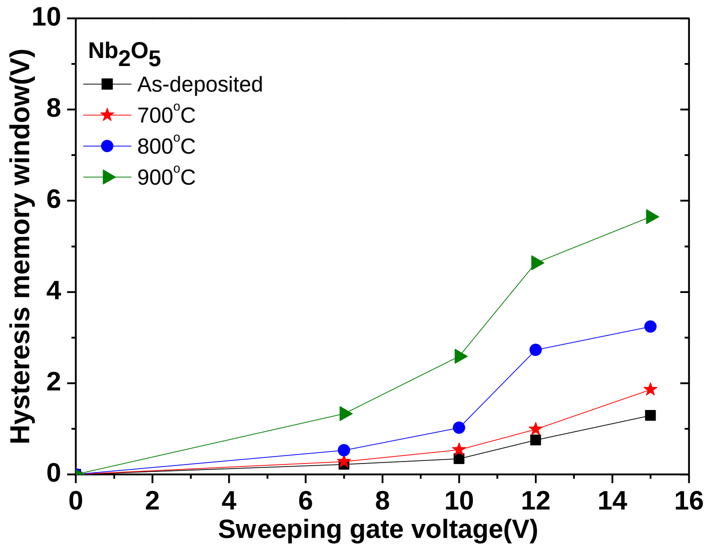

(a)

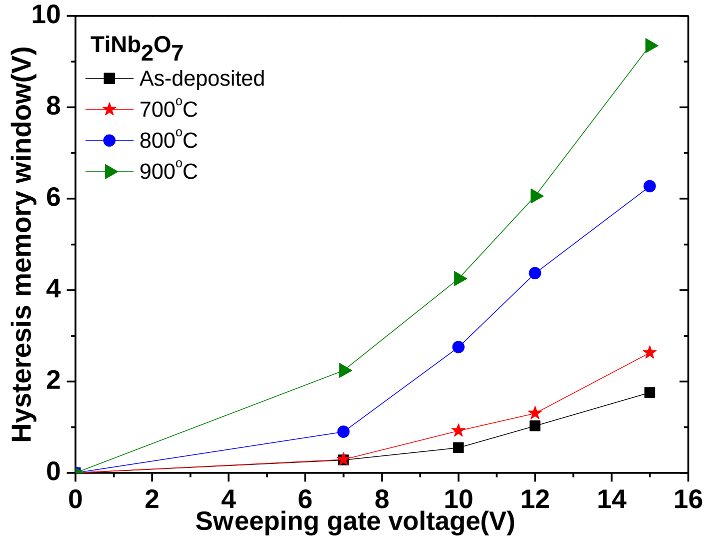

(b)

Figure S1. (a) Nb_2O_5 charge trapping nano-layers for different temperature after applying various bias.

(b) $TiNb_2O_7$ charge trapping nano-layers for different temperature after applying various bias.

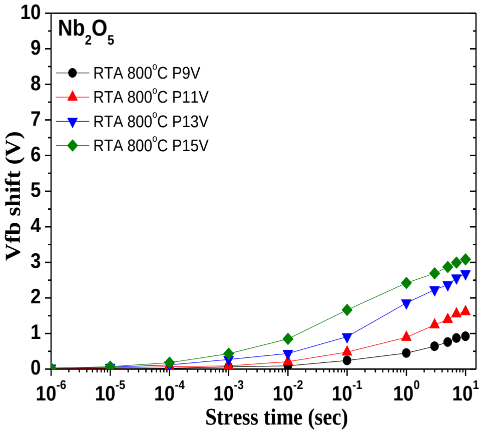

(a)

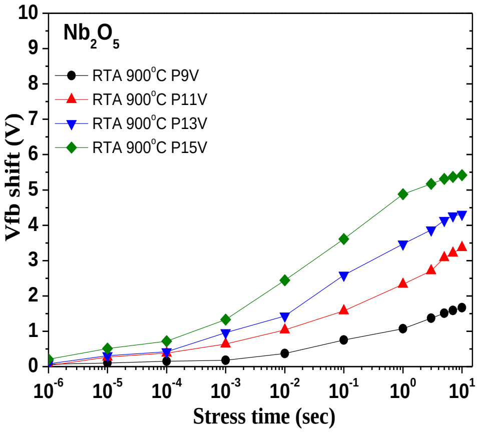

(b)

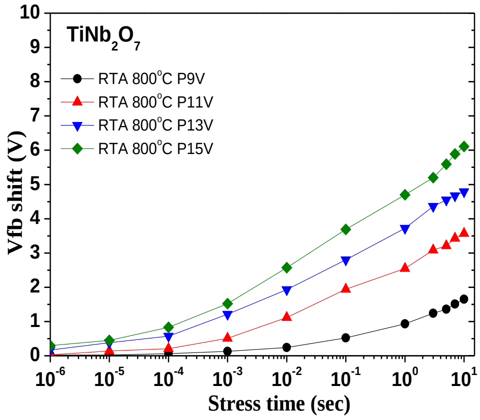

(c)

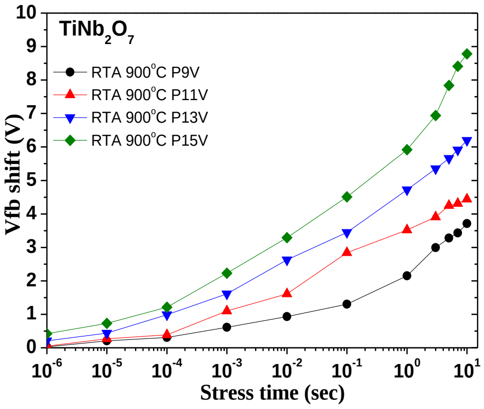

(d)

Figure S2 Programming speed of Nb_2O_5 trapping layer for (a) 800°C, and (b) 900°C after applying various bias.
Programming speed of $TiNb_2O_7$ trapping layer for (c) 800°C, and (d) 900°C after applying various bias.

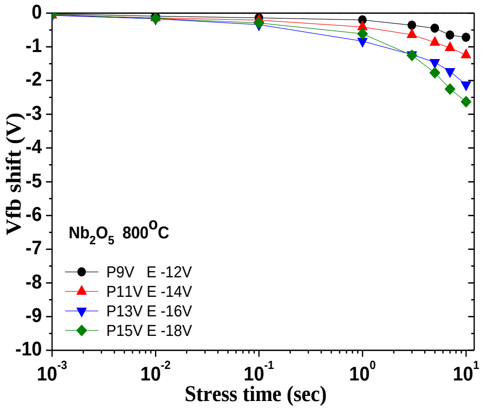

(a)

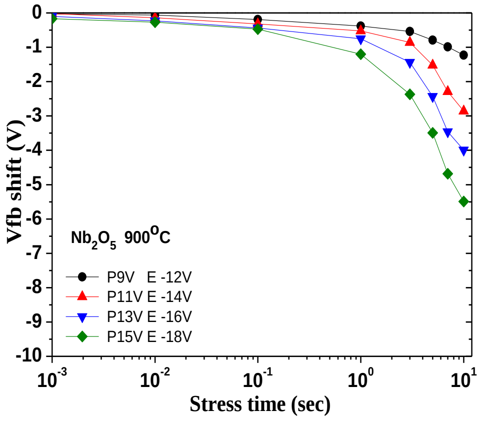

(b)

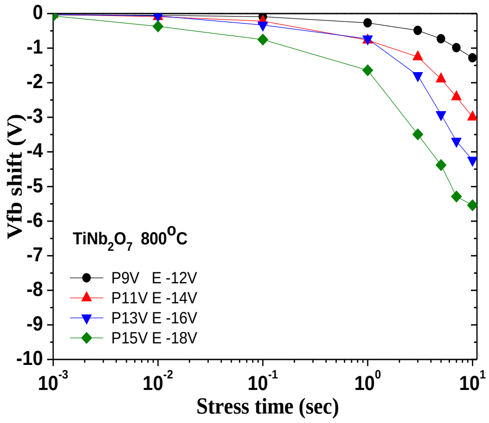

(c)

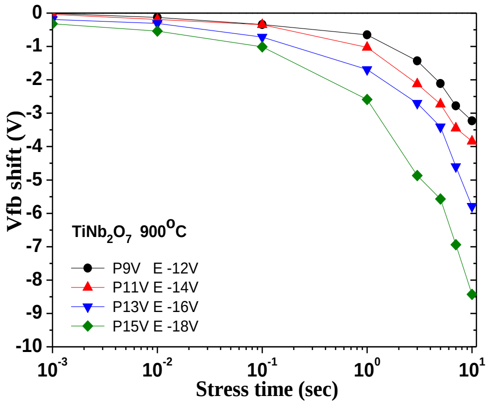

(d)

Figure S3 Erasing speed of Nb_2O_5 trapping layer for (a) 800°C, and (b) 900°C after applying various bias.
Erasing speed of $TiNb_2O_7$ trapping layer for (c) 800°C, and (d) 900°C after applying various bias.
